# SOCIAL INTERACTIONS AND WELL-BEING: THE ROLE OF COMMUNICATION METHOD

**DOI:** 10.1093/geroni/igz038.700

**Published:** 2019-11-08

**Authors:** Xin Yao Lin, Xin Yao Lin, Margie E Lachman

**Affiliations:** Brandeis University, Waltham, Massachusetts, United States

## Abstract

Given the increased usage of technology for social interactions, it is important to consider whether the method of communication makes a difference in one’s daily well-being. We conducted a diary study, over seven days with 142 participants, to examine the role that communication method (in-person, phone, technology) plays in daily stress exposure, stress reactivity, and positive and negative affect among adults ages 22 to 94. Multilevel modeling results revealed that on days with higher use of technology (text, video, internet) communication than their weekly average, individuals had more negative outcomes (greater stress exposure and negative affect). On days with more in-person communication than their weekly average, individuals had more positive affect. On days with more phone communication than their weekly average, individuals had less negative affect. The discussion highlights the benefits of in-person and phone communications for well-being, while also considering the potential negative outcomes associated with frequent technology communication.

